# Prediction of train wheel diameter based on Gaussian process regression optimized using a fast simulated annealing algorithm

**DOI:** 10.1371/journal.pone.0226751

**Published:** 2019-12-30

**Authors:** Xiaoying Yu, Hongsheng Su, Zeyuan Fan, Yu Dong

**Affiliations:** 1 College of Automation and Electrical Engineering & Key Laboratory of Opto-Technology and Intelligent Control Ministry of Education, Lanzhou Jiaotong University, Lanzhou, Gansu, China; 2 School of Rail Transportation, Shandong Jiaotong University, Jinan, Shandong, China; Nanyang Technological University, SINGAPORE

## Abstract

An algorithm to predict train wheel diameter based on Gaussian process regression (GPR) optimized using a fast simulated annealing algorithm (FSA-GPR) is proposed in this study to address the problem of dynamic decrease in wheel diameter with increase in mileage, which affects the measurement accuracy of train speed and location, as well as the hyper-parameter problem of the GPR in the traditional conjugate gradient algorithm. The algorithm proposed as well as other popular algorithms in the field, such as the traditional GPR algorithm, and GPR algorithms optimized using the artificial bee colony algorithm (ABC-GPR) or genetic algorithm (GA-GPR), were used to predict the wheel diameter of a DF11 train in a section of a railway during a period of major repairs. The results predicted by FSA-GPR was compared with other three algorithms as well as the real measured data from RMSE, MAE, R^2^ and Residual value. And the comparisons showed that the predictions obtained from the GPR optimized using FSA algorithm were more accurate than those based on the others. Therefore, this algorithm can be incorporated into the vehicle-mounted speed measurement module to automatically update the value of wheel diameter, thereby substantially reducing the manual work entailed therein and improving the effectiveness of measuring the speed and position of the train.

## Introduction

With the rapid development of high-speed railways worldwide, the driving speeds of trains continue to increase [1,2]. To ensure safety, train control systems must exhibit greater speed measurement accuracy than ever before. The speed and position information of a train are very important parameters to ensure normal operations of the train control system and safe operation of the train[3,4]. If the measured value of train speed is less than the actual value, there is a risk of rear-end collision, which affects driving safety. If the measured value of train speed is greater than the actual value, it leads to early braking and affects driving efficiency. Therefore, improving the measurement accuracy of the speed and position of a train is essential for driving safety and efficiency.

The most widely used speed measurement device in current train-mounted vehicle speed measuring modules is the axle speed sensor [5]. The calculation accuracies of speed and distance are affected by the reduction in wheel diameter and pulse counting error resulting from the idling and slipping of the train wheel.

Researchers in related fields have presented a few solutions aimed at cumulative pulse counting error compensation. At present, auxiliary sensors are often used to correct the problem of the wheel diameter decreasing gradually owing to trailing distance accumulation, but this method entails a few problems. For instance, using the method of multi-sensor information fusion to realize train wheel diameter correction increases the calculation load of the system to a certain extent, and the calibration fails once the sensor fails [6]. Using the exact distance between two adjacent transponders to achieve wheel diameter correction is expensive, increases maintenance and repair workload, and is also detrimental to the latest update of the line [7]. Using global positioning system to correct the wheel diameter value would cause large errors [8]. Odometer readings and acceleration detection threshold values have been used in the literature to compensate for the measurement errors caused by idling, skidding, and reduced diameter wear of the wheels. The Gaussian process was applied to short-term traffic flow prediction, motion trajectory prediction, and sample affinity prediction and classification of cell body type 4 domains/peptide system number in the literature [9–11], and good results were obtained. The common conjugate gradient method was used to determine the optimal hyper-parameters during the Gaussian process, but this method has a few defects: difficulty in determining the iterations, strong dependence of the initial value, and easier access to the local optimum rather than global master defects. Hence, a GA was proposed [12] and the firefly colony optimization algorithm was applied to determine the hyper-parameters of the Gaussian process [13], but both methods have disadvantages such as population degradation and low optimization speed. A combination of the inertial sensor and odometer has been proposed for positioning [14] to realize the calibration of problems such as diameter reduction, idling, and skidding of the wheel to reduce calculation errors and improve the measurement accuracy of positioning and speed. In another study, idling or sliding was detected by setting the threshold value of acceleration detection, and the velocity value was compensated for by using the linear interpolation method in the state of idling [15].

The Gaussian process is a new nonlinear machine learning method developed in recent years following neural networks and support-vector machines, and is based on the Bayesian network theory [16, 17]. It has strong reasoning ability, good interpretability, and exhibits strong adaptability in case of issues such as high dimensions, small samples, and nonlinearity. Furthermore, it has the advantages of no function constraint, adaptive acquisition of model parameters, and probabilistic output, which is a research hotspot in the field of machine learning [18]. The conjugate gradient method is generally used to determine the optimal hyper-parameters of the Gauss process [19]. This method has a few defects, such as difficulty in determining the number of iterations, strong dependence on the initial value, and greater ease of obtaining the local optimal than the global optimal. To solve these problems, a method based on an FSA algorithm to optimize GPR in the prediction of train wheel diameter is presented in this paper; by studying the historical wheel measurement data, the relationship between train wheel diameter value and travel distance can be researched with the objectives of correcting the wheel diameter value during train operation and reducing the measurement error.

Although there are many studies on the prediction of train wheel diameter, there are few based on field data. Therefore, in this study, we establish a train wheel diameter prediction model that is based on wheel data measured in the field and uses GPR optimized by FSA algorithm to determine the dynamic relationship between the wheel diameter value and running distance of the train with the objective of realizing real-time correction of the wheel diameter value in the process of train operation.

The speed measurement sensor characteristics of speed sensor and wheel tread are described in section "Materials and methods". At the same time, the specific method and realization process of train wheel diameter prediction with FSA-GPR method are expounded in this part. Then, the method proposed in this paper is used to simulate the wheel diameter of DF11 type locomotive in section “Simulation”. Finally, the predicted data and comparison results are presented in section “Results and discussion”, and the conclusion is given at the end of the paper.

## Materials and methods

### Analysis of speed measurement characteristics of speed sensor and wheel tread

The travel speed and travel distance of the train are calculated by the speed sensor according to the number of wheel revolutions recorded in a certain period of time and the stored value of the wheel diameter. Currently, the commonly used types of sensors are Hall-type pulse sensors and photoelectric speed sensors, both of which are installed on the train wheel axle but have different pulse counting methods. The formulae for calculating the train speed *v* and distance Δ*s* are as follows:

$$v=\frac{\pi \times D\times f_{m}}{p}$$ (1)

$$\Delta s=\frac{n\times \pi \times D}{p}$$ (2)

In these formulae, *p* is the number of pulse outputs of the sensor per revolution of the wheel, *f*_*m*_ is the output frequency of the speed sensor, *D* is the wheel diameter value, and *n* is the pulse measurement value during this period.

It can be seen from formula (1) and (2) that the wheel diameter value is an important parameter in calculating the speed and distance of the train. However, as train mileage increases, the wheel diameter becomes smaller because of wear. If the wheel diameter value pre-stored on the on-board computer cannot be calibrated in time, the calculation error increases and the positioning error gradually accumulates as the train runs, thereby affecting driving safety and efficiency.

**Fig 1:**
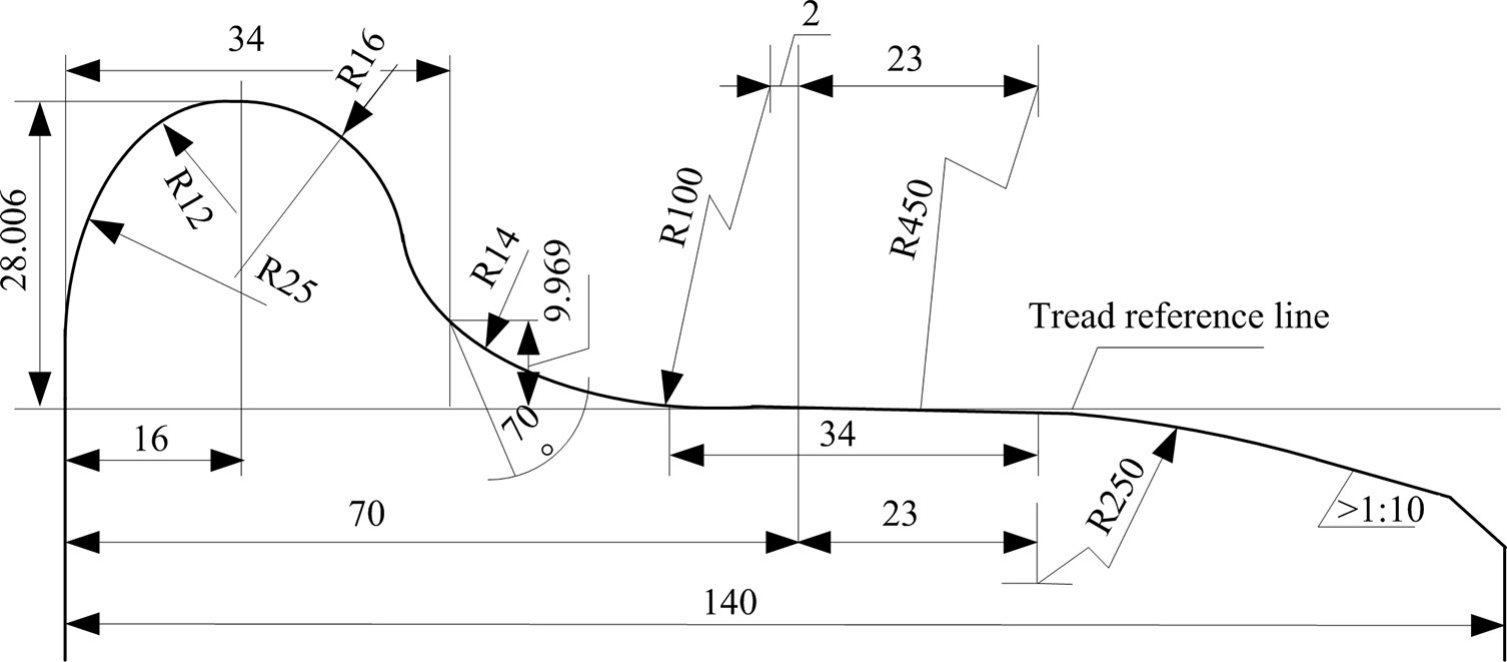
JM3 type tread shape.

According to the provisions of the Transport Department of the Ministry of Railways Transportation Bureau (2001235), the JM3 type of treads should be used in new and overhauled locomotives effective January 2001, the dimensions are shown in Fig 1.

The point on the tread surface, which is 70 mm from the inside of the wheel, is defined as the base point, and the circle formed by the base point along the circumference of the wheel is called the rolling circle [20]. At present, the diameter of the rolling circle is manually measured using a wheel diameter gauge on the spot, and the tread wear is obtained by subtracting the current wheel diameter from the wheel diameter stored in the standard system. However, as long as the train is running, the wheels continue to be worn down and their diameters continue to change. In reality, the work of obtaining wheel-related data measurements are undertaken every two or three months, during which the change in wheel diameter during train operations is not corrected, resulting in a difference between the calculated train speed and its actual value. The wheel wear rate is normally distributed and there is a nonlinear mathematical relationship between wheel wear and driving distance [21]. This indicates that there is a nonlinear mathematical relationship between wheel diameter and train mileage. Therefore, in this study, we adopted an intelligent learning algorithm to analyze and model the relevant data obtained from the travel distance recorded monthly when the train runs in the sections and thus determine the nonlinear mathematical relationship between the train wheel diameter value *y*, and train travel distance *x*, so that the wheel diameter value at any time can be predicted in advance based on the travel distance.

### GPR theory

Predicting the current wheel diameter size based on the actual running distance of the train is a process of nonlinear function value estimation and extrapolation. The Gaussian process is a new nonlinear machine learning method developed in recent years following neural networks and support-vector machines; it is a Bayesian inference from the perspective of function space to describe the function distribution [22]. According to the Bayesian theory, posterior knowledge is obtained by correcting prior knowledge using sample information, i.e.

p(f(x)|D)∝p(y|f(x),X)p(f(x)) (3)

In the formula (3), *p*(*f*(*x*)) is the prior knowledge, and *p*(*f*(*x*)|*D*) is the posterior knowledge, i.e., the current knowledge, and *p*(*y*|*f*(*x*),*X*) indicates sample information. The Gaussian process supposes a set of random variables *f*(*x*), and its arbitrary finite number of random variables satisfies the joint Gaussian distribution [23]. Then

$$f(x)∼GP(m(x),k(x,x^{'})),$$ (4)

where *m*(*x*) is the mean value, and *k*(*x*,*x*') is the covariance. In general, the mean function is 0, where ***x*** and *x*'∈*R*^*d*^ are arbitrary random variables. In the regression problem, the model for the input training data set $D={\left\{\left(x_{i},y_{i}\right)\right\}}_{i=1}^{n}=(x,y)$ is as follows.

y=f(x)+ε, (5)

where ***x*** is the input vector, *f*(*x*) is the unknown function, and *y* is the corresponding output scalar affected by noise; it is assumed that the noise *ε* obeys a normal distribution with a mean value of 0 and a variance of $\delta_{n}^{2}$. The key hypothesis of the GPR is that, the values of *x* are given, *y* is to be modeled, and the corresponding values of *y* obey the joint normal distribution. If two values of *x* are similar, then the corresponding *y* values are similar too, i.e., the covariance matrix is a function of *x*. Then the prior distribution of the observed values of *y* is

$$y∼N(0,K(X,X)+\delta_{n}^{2}I_{n})$$ (6)

In the formula (6), *K*(*X*,*X*) = *k*(*x*_*i*_,*x*_*j*_)_*n*×*n*_ is a positive definite covariance matrix, and any terms of the matrix represent the relevance of *x*_*i*_,*x*_*j*_, *I*_*n*_ is a unit matrix.

The train travel distance x*as a new input value is used to predict the wheel diameter output value y*; when new points are added to the above random variable, a joint distribution can be obtained as follows.

$$\left(\begin{array}{c} y\\ y^{*} \end{array}\right)∼\left(0,\left[\begin{array}{cc} K(X,X) & K(X,x^{*})\\ K(x^{*},X) & k(x^{*},x^{*}) \end{array}\right]\right)$$ (7)

In the formula, *K*(*X*,x*) is the n × 1-order covariance matrix between the training set ***X*** and new point x^*^, and *k*(x*,x*) is the auto-covariance of the new point x*. According to Bayesian theory, the posterior distribution of y* can be obtained from Eq (7), which also obeys the Gaussian distribution.

$$y^{*}∼N(m(x^{*}),v(y^{*}))m(x^{*})=K(x^{*},X){\left[K(X,X)+\delta_{n}^{2}I_{n}\right]}^{-1}y$$ (8)

$$v(y^{*})=k(x^{*},x^{*})-K(x^{*},X){\left[K(X,X)+\delta_{n}^{2}I_{n}\right]}^{-1}K(X,x^{*})$$ (9)

In the Eqs (8) and (9), *m*(x*) is the mean value and *v*(y*) is the variance estimation results of the predicted values corresponding to the new point x* [24].

For the training of the GPR model, the square index kernel function is used as the covariance function in this study, i.e.

$$k(x,x^{'})=\delta_{f}^{2}\mathrm{esp}(-\frac{1}{2}{\left(x-x^{'}\right)}^{T}M^{-1}(x-x^{'})),$$ (10)

where $\delta_{f}^{2}$ is the signal variance of the covariance function used to control the degree of local correlation, *M* = *diag*(*l*^2^) is a diagonal matrix of hyper-parameters, and *l* is a measure of variance. Then, the set of hyper-parameters, $\theta =\left\{l,\delta_{f}^{2},\delta_{n}^{2},\right\}$, of the GPR model can be obtained.

Generally, based on the input training data set, the maximum likelihood function method is used to obtain the set of hyper-parameters *θ*. After determining *θ*, the mean *m*(x*) and variance *v*(y*) can be obtained by using Eqs (8) and (9) respectively, and the prediction can be performed according to the GPR model determined. However, the method of obtaining the hyper-parameters is the key to the application of the Gaussian process. In view of the shortcomings of the current conjugate gradient method, we used the FSA algorithm to obtain the optimal hyper-parameters in this study.

### Optimization of GPR by FSA algorithm

At present, the widely used global optimization algorithms include simulated annealing (SA) algorithms, group intelligence algorithms, GAs, and other bionic intelligent algorithms developed by simulating natural phenomena. The FSA algorithm is not only superior to GA and group intelligent algorithm in terms of accuracy and speed of optimization results, but also has a simple calculation process, wide application range, and strong global optimal determination ability. The SA algorithm is a heuristic random search process of the Monte Carlo iterative method [25], which adopts the Metropolis receiving criterion and the global optimization method with probability 1 convergence. However, it must have a strict annealing plan to obtain the global optimum. When the initial temperature is adequately high, the annealing speed is slow, the number of disturbances at the same temperature is large, the number of iterations is large, and convergence speed is too low. Aiming at ensuring the efficiency of the SA solution, we used the FSA algorithm in this study to optimize the parameter solving problem of the Gaussian process and realize wide ranges of fast search and partial fine search. The FSA algorithm ensures the performance of global optimization in a limited time by improving the model disturbance mode, acceptance probability, and annealing mode of the SA.

Model perturbation: Simulated annealing usually uses the Gaussian distribution method to perturb the current model to generate a new model. The algorithm uses a temperature-dependent Cauchy distribution to perturb, i.e.

$$M_{i}^{\prime }=M_{i}+y_{i}(B_{i}-A_{i})$$ (11)

$$y_{i}=T[\mathrm{sgn}(u-0.5)[{\left(1+1/T\right)}^{\left|2u-1\right|}-1],$$ (12)

where *M*_*i*_ is the *i*^*th*^ variable of the current model, $M_{i}^{\prime }$ is the *i*^*th*^ variable of the new model after the disturbance, [*A*_*i*_,*B*_*i*_] is the range of values of *M*_*i*_ and $M_{i}^{\prime }$, sgn(X) is a sign function, and *u* is a uniformly distributed random number in the range [0, 1].

Acceptance probability: The new acceptance probability is calculated according to the generalized Gibbs distribution.

$$P={\left[1-(1-h)\Delta E/T\right]}^{1/(1-h)},$$ (13)

where Δ*E* = *E*(*m*')−*E*(*m*) is the difference in values between the new model objective function generated after the disturbance and the current objective function, *h* is a real number, and *T* is temperature. When the value of *h* approaches 1, the acceptance probability formula of the conventional SA can be obtained, as represented by Eq (14), which is a special case of Eq (13).

P=exp(−ΔE/T) (14)

Annealing method:

$$T(k)=T_{0}\alpha^{k^{1/N}},$$ (15)

where α∈(0.7,1.0) is usually the temperature attenuation rate, *T*_0_ is the initial temperature, *k* is the number of iterations, and *N* is the number of parameters to be inverted. In practical applications, 1/*N* is usually replaced with 1 or 0.5.

### Wheel diameter prediction method

**Fig 2:**
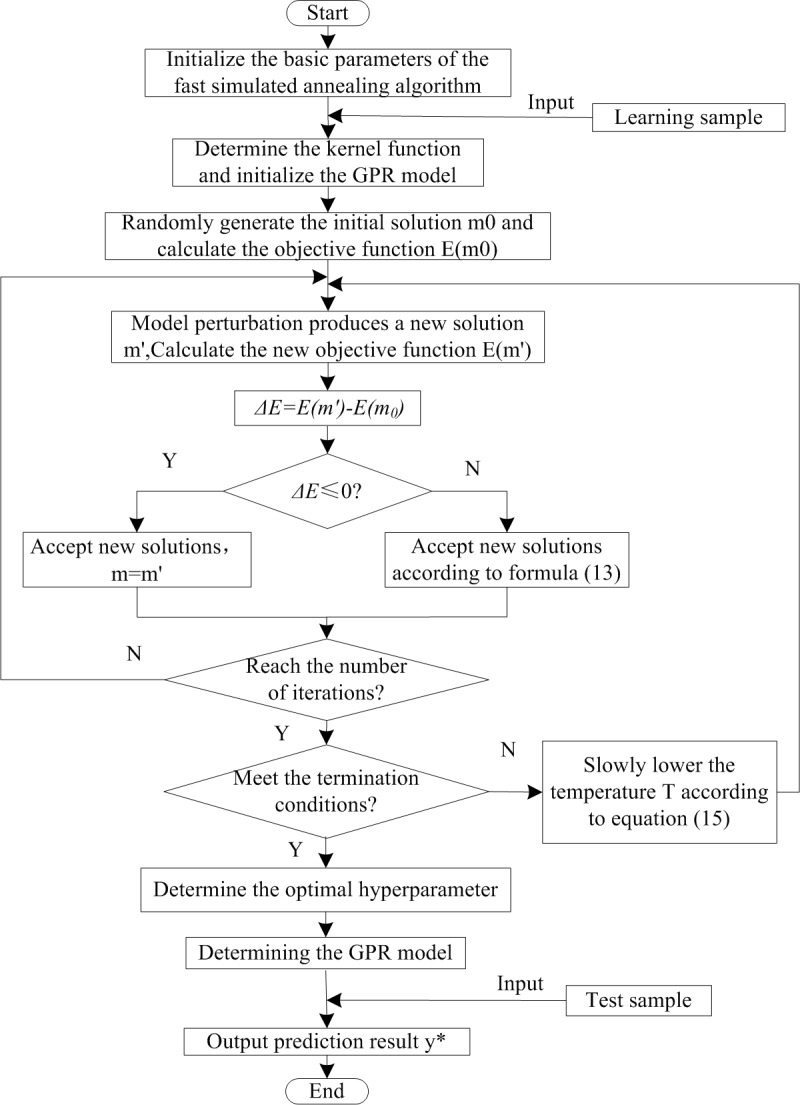
FSA optimized GPR prediction algorithm flow.

In this study, the FSA algorithm is applied to the learning of GPR, and the hyper-parameters are intelligently optimized. Starting from the initial solution, slow cooling is conducted under the control limits of the temperature control parameters, each of which is taken through an iterative process of disturbing to generate a new solution, calculating the energy function *E*(*m*), judging, and accepting or discarding a new solution, following which the optimal solution is determined, and the optimal set of hyper-parameters is returned to determine the GPR model. The value of the input train running distance is then predicted, and the output, i.e., the predicted value of the train wheel diameter at this time is obtained. The flow chart of the prediction algorithm is shown in Fig 2.

The specific steps are as follows:

1. Divide the sample set into a training sample set *D* and a test sample set D*. The algorithm inputs are *D* and the value of x* of the set D*, and the output is the target value y* of the set D*.
2. Initialize the basic parameters of the SA algorithm, including the initial temperature *T*_0_, initial solution *m*_0_, *T*_*i*_ (the next temperature value of *T* in the cooling schedule), and the number of iterations *k*.
3. Select the squared exponential covariance as the kernel function, and initialize the GPR model.
4. Calculate the initial objective function *E*(*m*_0_), then perturb the model to generate a new solution *m*', and calculate its corresponding *E*(*m*') and Δ*E* = *E*(*m*')−*E*(*m*_0_).
5. If Δ*E*≤0, accept the new solution *m* = *m*', otherwise accept the new solution according to Eq (13).
6. Determine whether the number of iterations *k* is exceeded at temperature *T*_*i*_. Repeat steps (4) and (5) if it is not exceeded. If it is exceeded, then judge whether *T*_*i*_ reaches the termination temperature *T*_*Z*_. If it does, the optimal solution is the output, thereby determining the GPR model. Then, input the test data and output its corresponding prediction result y*. Else, continue to cool according to Eq (15) and return to step (4).

### Simulation

Normally, the wheel diameter of a locomotive is artificial measured every 20,000 kilometers. We extracted the real value of wheel diameter measurements of 29 DF11 type locomotive wheels provided by a locomotive depot of Harbin railway bureau for five times during an overhaul, in total 145 sets as the simulation data. Some of the data are shown in Table 1, the data reflect the relationship between train wheel diameter and kilometers the train traveled. The DF11 train wheel adopts the JM3 type tread. Its standard diameter is 1050^+2^_–1_ mm, and the minimum diameter limit is 975 mm. If the wheel diameter is less than 975 mm, it must be replaced with a new one. All the 29 wheels selected in this paper have not been replaced during the test period.

**Table 1:** DF11 locomotive wheel diameter measurement report (five times).

NO.	Measured value of wheel diameter (mm) / Mileage (km)				
	Measure 1	Measure 2	Measure 3	Measure 4	Measure 5
1	1051.43/3697	1047.12/22368	1040.14/297340	1032.85/365892	1029.19/380129
2	1051.65/17466	1040.24/229101	1030.65/356422	1026.67/413613	1003.72/579630
3	1050.25/6750	1042.16/256980	1030.97/385843	1022.09/455627	1001.19/533598
4	1044.52/236580	1040.23/298652	1036.45/329874	1029.48/383635	1018.49/483245
5	1017.24/509823	1008.42/530397	1006.44/562397	1003.27/580846	1000.32/602201
6	1051.12/6982	1044.13/297635	1036.15/326971	1031.10/369640	1001.19/490398
7	1016.82/403691	1010.08/537620	1006.65/556972	1003.45/579621	1001.93/598231

Among the 145 sets of relevant data, 35 sets were used as test samples. In regression, over-fitting may occur due to the complexity of model parameters, which can achieve good results in the training set but poor results in the test set [26]. In order to prevent the occurrence of the over-fitting phenomenon, the remaining 110 sets were randomly divided into 5 groups for 5-fold cross-validation. 4 of them were randomly selected as the training set each time, and the remaining 1 was used as the verification set. After one round of validation, another four groups were randomly selected to train the data. When 5 rounds finished, the loss function was selected to evaluate the optimal model and parameters.

To verify that the FSA-GPR algorithm proposed in this paper can effectively predict the train wheel diameter value, other algorithms, GPR, GA-GPR, and ABC-GPR were used with the same data set for the purposes of processing and comparison.

GPR can be directly used in probabilistic prediction because of its advantages such as easy realization, adaptive acquisition of hyper parameters and probabilistic of prediction results [12,23].

As a bionic optimization algorithm, GA can achieve global optimization by imitating the natural selection and genetic mechanism, it has been widely used to solve problems with multiple parameters, variables and objectives, and has achieved good application results [27].

ABC is a group intelligence algorithm based on global search and iterative optimization. It has the advantages of simple operation, easy implementation, strong adaptability, high search accuracy and strong robustness. It is outstanding in solving the optimization problem of function extremum [28, 29].

In the process of simulation, the four algorithms mentioned above were used to study the sample data to determine the prediction model, and the trained model was then used to predict the test set data. The parameter settings of each algorithm are shown in Table 2.

**Table 2:** Parameter setting situation.

Algorithm name	Main parameter setting
**Fast simulated annealing algorithm (FSA)**	*α* = 0.96, *N* = 1, The initial temperature *T*_0_ = 200, Number of disturbances per temperature *k* = 3
**Genetic algorithm (GA)**	The population size is 30, the probability of hybridization is 0.85, the probability of mutation is 0.02, and the evolutionary algebra is 100.
**Artificial bee colony algorithm (ABC)**	Number of honey sources *S*_*N*_ = 20, number of iterations *T* = 100, maximum number of times of honey source mining *Limit* = 100

## Results and discussion

**Fig 3:**
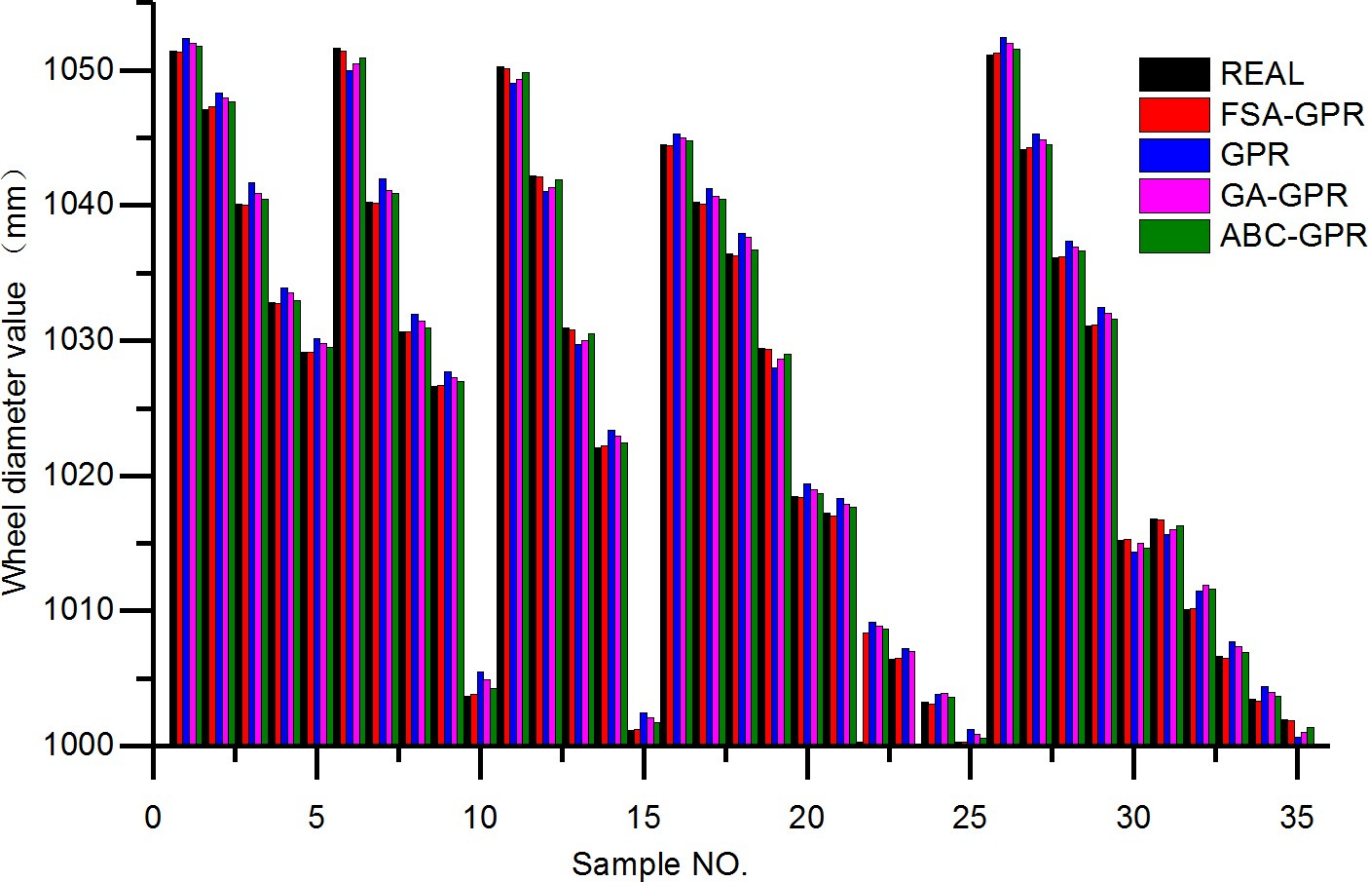
Comparison of wheel diameter value prediction results of four algorithms.

The prediction results of the above four algorithms on the train wheel diameter values of 35 test sets are shown in Fig 3. It can be intuitively seen from the results that the predicted results of the FSA-GPR algorithm used in this paper are closest to the actual measured values, while the predicted values of the other three algorithms are greatly deviated from the actual values.

Three indicators, RMSE, MAE and the coefficient of determination *R*^*2*^ are defined as the evaluation factors to compare the predicted effects of each method intuitively. The calculation formulae are as follows:

$$RMSE=\sqrt{\frac{\sum_{i=1}^{n}(y_{i}^{\prime }-y_{i}{)}^{2}}{n}}$$ (16)

$$MAE=\frac{1}{n}\sum_{i=1}^{n}\left|y_{i}^{\prime }-y_{i}\right|$$ (17)

$$R^{2}=1‐\frac{\sum_{i=1}^{n}(y_{i}-{\hat{y}}_{i}{)}^{2}}{\sum_{i=1}^{n}(y_{i}-\bar{y}{)}^{2}}$$ (18)

where *n* is the number of predicted samples, $y_{i}^{'}$ is the predicted value of the output of the *i*^*th*^ predicted sample, *y*_*i*_ is the true value of the *i*^*th*^ predicted sample, and $\bar{y}$ is the mean value of the sample. ${\hat{y}}_{i}$ is the sample regression fitting value.

A comparison of the prediction indices of the four algorithms is given in Table 3, It can be seen from it that the RMSE value obtained by the proposed prediction method is reduced by 0.7803, 0.3414, and 0.2188 than the others respectively, and from the table, we can see that the MAE of FSA-GPR is the lowest one, it also shows that the predicted value by FSA-GPR is closest to the real measured value, the coefficient of determination *R*^*2*^ of the FSA-GPR is the closest to 1. The closer *R*^*2*^ is to 1, the closer the prediction point is to the sample one, and the better the fitting effect is. All the results above verify the feasibility of the algorithm proposed in this paper.

**Table 3:** Comparison of four algorithm prediction indicators.

Algorithm	RMSE	MAE	R^2^
**GPR**	0.9137	0.7623	0.7356
**GA-GPR**	0.4748	0.4503	0.8601
**ABC-GPR**	0.3522	0.2771	0.9012
**FSA-GPR**	0.1334	0.1251	0.9634

In order to visually observe the relationship between the predicted results of four algorithms on the wheel diameter of 35 test sets and the actual measured values, the concept of residual is introduced here. Residual value *e*_*i*_ is an important indicator to investigate the adaptability of a regression model to the given data, as shown in Eq (19).

$$e_{i}≙y_{i}-y_{i}^{'}(i=1,2,\ldots ,n),$$ (19)

**Fig 4:**
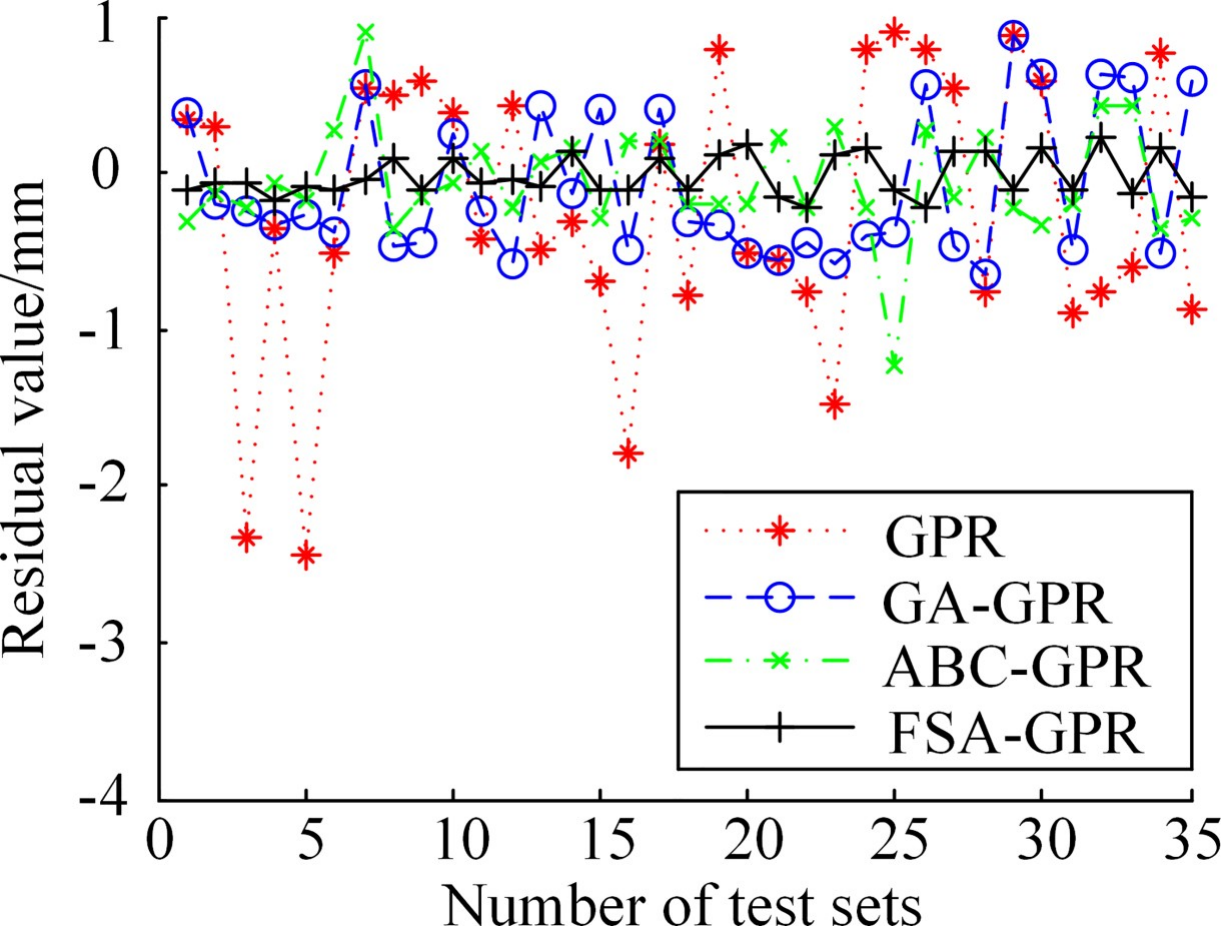
Residual comparisons of the four algorithms.

The residual value can visually reflect the degree of deviation between the predicted and real measured wheel diameter values. The smaller the residual value, the closer the predicted value is to the real measured one and the better the prediction effect is. The residual diagram of the predicted results of the four algorithms is shown in Fig 4.

It can be seen from Fig 4 that, the unoptimized GPR method had the worst performance in wheel diameter prediction, its prediction results showed asymmetry and some abnormal extremum points. For example, at the 3rd, 5th, 16th and the 23rd prediction points, and the prediction results had a large deviation. The same problem existed in the ABC-GPR and GA-GPR methods, only slightly better than the GPR method. However, the FSA-GPR method proposed in this paper presented a symmetric state and the trend of variation in the train wheel diameter value can be better tracked. The residual value of the effect of the prediction of the FSA-GPR algorithm proposed in this paper is more concentrated than those of the other three, and the fluctuation range of the residual variation is relatively small, which is closest to the real measured value.

As can be seen from the above comparison results of RMSE, MAE and residual value of the prediction results, The simulation effects of the above four algorithms are sorted as follows, FSA-GPR, ABC-GPR, GA-GPR and GPR.

The task of these optimization algorithms is to search for the optimal hyper-parameters of improved GPR. In the process of optimization model, the advantages and disadvantages of each algorithm are also reflected. The traditional GPR algorithm adopts conjugate gradient method to improve the optimal hyper-parameters. This method has a strong dependence on the initial value and is easy to fall into local optimization. The GA-GPR method does not rely on the initial value and can achieve global optimization. However, experiments show that GA is relatively weak in searching for improved hyper-parameters after more than 6000 times of evaluation. ABC-GPR is an iterative optimization process, which is based on global search and has a strong robustness. However, its search time is relatively long. When the sample data and the evaluations number are large, its time-consuming disadvantage becomes more obvious. FSA-GPR does not need to calculate the derivative of the function model equation, which avoids the problem of difficult derivative of complex functions, it realizes global search, and avoids falling into local optimization, this method has the best comprehensive performance in solving the wheel diameter prediction problem proposed.

## Conclusions

In this paper, an FSA algorithm is used to optimize GPR in the prediction of train wheel diameter. Following case verification and comparative analysis, the following conclusions can be drawn.

In the case of small samples and nonlinear problems like Prediction of train wheel diameter proposed in this paper, FSA-GPR has achieved good prediction results, both its RMSE and MAE were lower than other algorithms, and its R^2^ was closest to 1, which renders it suitable for the prediction of train wheel diameter.

An FSA algorithm is adopted to optimize the solution of the Gaussian process hyper-parameter problem. This has several advantages. It can effectively avoid the disadvantages of the traditional conjugate gradient method, improve the convergence speed, and quickly search for the global optimal solution, thereby improving the generalization ability of GPR.

The FSA-GPR algorithm proposed in this paper achieved better results than those of the other algorithms in the prediction of wheel diameter, and its effectiveness and accuracy were verified.

## Supporting information

S1 Table DF11 locomotive wheel diameter measurement report (145sets). (PDF)
