# Supplementary material for: Prediction of train wheel diameter based on Gaussian process regression optimized using a fast simulated annealing algorithm
Source: PLoS One. 2019 Dec 30;14(12):e0226751. doi: 10.1371/journal.pone.0226751 (PMC6936821; doi:10.1371/journal.pone.0226751)
Supplement: S1 Table — (PDF) [file pone.0226751.s001.pdf]

Table 1. DF11 locomotive wheel diameter measurement report (145sets)

Wheel NO.	Measured value of wheel diameter (mm) / Mileage (km)				
	Measure 1	Measure 2	Measure 3	Measure 4	Measure 5
1	1051.43/3697	1047.12/22368	1040.14/297340	1032.85/365892	1029.19/380129
2	1051.65/17466	1040.24/229101	1030.65/356422	1026.67/413613	1003.72/579630
3	1050.25/6750	1042.16/256980	1030.97/385843	1022.09/455627	1001.19/533598
4	1044.52/236580	1040.23/298652	1036.45/329874	1029.48/383635	1018.49/483245
5	1017.24/509823	1008.42/530397	1006.44/562397	1003.27/580846	1000.32/602201
6	1051.12/6982	1044.13/297635	1036.15/326971	1031.10/369640	1001.19/490398
7	1016.82/403691	1010.08/537620	1006.65/556972	1003.45/579621	1001.93/598231
8	1048.06/166892	1034.65/322492	1030.36/356825	1027.67/408743	1011.63/482210
9	1032.86/33458	1021.03/425290	1006.01/486321	995.05/601330	992.81/635448
10	1048.04/9652	1041.44/298611	1036.96/332016	1024.83/453622	1018.20/504698
11	1024.07/459870	1011.05/542095	998.01/604698	989.01/660789	983.06/698315
12	1046.24/298762	1030.41/367892	1026.04/432168	1014.28/482358	999.21/568164
13	1044.07/236510	1036.16/326751	1029.03/385697	1014.37/498264	1003.08/551328
14	1013.68/520364	1003.08/569821	992.00/645832	984.03/678120	979.66/705698
15	1048.27/85980	1041.36/262684	1030.27/375695	1017.55/465982	1000.27/584002
16	1015.44/486253	1006.08/554800	999.03/598763	986.03/653921	978.40/786952
17	1032.64/365980	1023.43/465308	1009.47/536980	996.48/639403	983.40/682975
18	1025.04/403689	1012.06/523640	1005.15/552907	997.04/635948	982.06/675621
19	1049.80/30268	1040.41/259842	1031.03/365497	1018.27/486892	1003.85/587623
20	1050.04/25624	1043.04/220368	1036.06/334213	1021.04/465972	1003.02/552873
21	1045.84/220354	1033.08/358761	1022.15/496501	1005.82/586231	986.83/669035
22	1047.01/213654	1034.64/336597	1020.03/486932	1008.36/553678	998.26/635297
23	1046.85/225691	1023.74/425932	1016.41/496500	1007.82/587798	993.83/654238
24	1038.3/303698	1030.2/386852	1017.3/498652	1006.3/598632	982.3/675600
25	1033.2/356892	1020.1/488562	1008.7/569820	992.4/645698	981.2/678902
26	1043.64/245698	1033.74/356982	1013.35/495632	1000.64/596210	986.60/660231
27	1045.61/256981	1032.64/360710	1021.69/462530	1010.67/536411	997.65/602354
28	1021.81/463298	1010.82/536210	1001.84/598621	993.86/659842	979.81/697210
29	1050.63/3250	1041.34/265984	1030.47/389651	1019.66/504163	1000.07/593642
